# Readability Formulas and User Perceptions of Electronic Health Records Difficulty: A Corpus Study

**DOI:** 10.2196/jmir.6962

**Published:** 2017-03-02

**Authors:** Jiaping Zheng, Hong Yu

**Affiliations:** ^1^ College of Information and Computer Sciences University of Massachusetts Amherst, MA United States; ^2^ Bedford Veterans Affairs Medical Center Center for Healthcare Organization and Implementation Research Bedford, MA United States; ^3^ Department of Quantitative Health Sciences University of Massachusetts Medical School Worcester, MA United States

**Keywords:** electronic health records, readability, patients

## Abstract

**Background:**

Electronic health records (EHRs) are a rich resource for developing applications to engage patients and foster patient activation, thus holding a strong potential to enhance patient-centered care. Studies have shown that providing patients with access to their own EHR notes may improve the understanding of their own clinical conditions and treatments, leading to improved health care outcomes. However, the highly technical language in EHR notes impedes patients’ comprehension. Numerous studies have evaluated the difficulty of health-related text using readability formulas such as Flesch-Kincaid Grade Level (FKGL), Simple Measure of Gobbledygook (SMOG), and Gunning-Fog Index (GFI). They conclude that the materials are often written at a grade level higher than common recommendations.

**Objective:**

The objective of our study was to explore the relationship between the aforementioned readability formulas and the laypeople’s *perceived* difficulty on 2 genres of text: general health information and EHR notes. We also validated the formulas’ appropriateness and generalizability on predicting difficulty levels of highly complex technical documents.

**Methods:**

We collected 140 Wikipedia articles on diabetes and 242 EHR notes with diabetes International Classification of Diseases, Ninth Revision code. We recruited 15 Amazon Mechanical Turk (AMT) users to rate difficulty levels of the documents. Correlations between laypeople’s perceived difficulty levels and readability formula scores were measured, and their difference was tested. We also compared word usage and the impact of medical concepts of the 2 genres of text.

**Results:**

The distributions of both readability formulas’ scores (*P*<.001) and laypeople’s perceptions (*P*=.002) on the 2 genres were different. Correlations of readability predictions and laypeople’s perceptions were weak. Furthermore, despite being graded at similar levels, documents of different genres were still perceived with different difficulty (*P*<.001). Word usage in the 2 related genres still differed significantly (*P*<.001).

**Conclusions:**

Our findings suggested that the readability formulas’ predictions did not align with perceived difficulty in either text genre. The widely used readability formulas were highly correlated with each other but did not show adequate correlation with readers’ perceived difficulty. Therefore, they were not appropriate to assess the readability of EHR notes.

## Introduction

### Background

Patient engagement and effective patient-physician communication are essential in patient-centered care, defined by the Institute of Medicine as “respectful of and responsive to individual patient preferences, needs, and values, and ensuring that patient values guide all clinical decisions” [1]. Electronic health records (EHRs) are a rich resource for developing applications to engage the patients and foster patient activation [2-4]. Thus, allowing patients access to their own EHR records holds a strong potential to enhance patient-centered care. It may improve the understanding of their own clinical conditions and treatments, leading to improved health care outcomes (eg, increased medication adherence [4]).

As patients express interests in reading their own EHR data [5], health care institutions have also begun to open up access to the EHR records [6]. However, EHRs are written by physicians to communicate with other health care professionals [7]. Therefore, EHRs are full of medical jargon, abbreviations, and other domain-specific usages and expressions that are ill-suited for the lay people (patients). One study showed that nearly two-thirds of the surveyed patients considered physicians’ notes difficult to understand, and radiology reports and nurses’ notes were also perceived as difficult [8]. Another study recruited healthy volunteers to read and retell medical documents [9]. Common retelling errors included misunderstanding clinical concepts and physician’s findings during a patient’s visit. In a study of electronic primary care records, many patients requested explanations of medical terms and abbreviations [10]. A recent patient survey on Web-based access to laboratory results concluded that test result comprehension still needed improvement [11]. Findings from an assessment of lay understanding of medical terms suggested that a substantial proportion of the lay public did not understand phrases often used in cancer consultations and that knowledge of basic anatomy could not be assumed [12]. In the emergency department setting, patients understood less than 30% of commonly used medical terms [13]. Moreover, the vocabulary gap between professionals and laypeople has motivated a thread of research to develop controlled vocabulary resources [14-16].

Merely providing patients with their own EHR records, therefore, does not necessarily help the patients better understand their own conditions. Further complicating the issue, it is estimated in the National Assessment of Adult Literacy that the average American has a reading level between the 7th and 8th grade [17]. It is also reported in the same assessment that about 36% of the US population or 75 million Americans have basic or below basic health literacy. The opaque narratives in the EHR present a challenge to the average patient.

### Electronic Health Records and Readability

Measuring the readability of the EHR notes is one important step toward making the notes accessible to the patients. Numerous studies [18-20] have evaluated the difficulty of health information intended for patient consumption using readability formulas. They conclude that the materials are often written at a grade level higher than common recommendations. However, the trust in these formulas to measure difficulty may be overextended. Grade-level readability formulas were originally developed to try to ensure that a school textbook for a particular grade was appropriate for children at that grade level [21]. Their capabilities in measuring documents of a highly technical nature such as health care are not thoroughly validated. There are recent attempts to develop methods for text in the medical domain [22,23]. They have yet to enjoy wide adoption in the community, which may be attributed to the fact that efforts in learning models are inevitable.

### Readability Formulas

Numerous readability metrics have been used for the purposes of preparing texts for schoolchildren and language learners and ensuring smooth written communication. These metrics assess the grade level or the number of years of education needed for a person to understand the content. Here we briefly introduce 3 of the metrics. For more discussions on these traditional readability formulas, we refer the reader to the review in [24].

Flesch-Kincaid Grade Level (FKGL) [25] predicts a grade level using the average sentence length and the average word length. Simple Measure of Gobbledygook (SMOG) [26] predicts readability based on the number of polysyllabic words (words with more than 3 syllables) and the number of sentences. Similarly, Gunning-Fog Index (GFI) [27] employs sentence length and the proportion of polysyllabic words. Detailed equations are shown in Multimedia Appendix 1.

These metrics are also used extensively in the health care domain to measure the readability of patient handouts [18,28-30], Web-based health information for patients [19,31,32], medication inserts [33,34], informed consent forms [20,35,36], clinical trial information [37], and Wikipedia medical entries [38,39]. FKGL, in particular, is used in more than half of readability studies compared in one review [40].

In general, these aforementioned metrics rely on the assumption that the longer the words and the sentences, the more difficult the text is. However, this assumption may not hold true for EHR narratives, which contain lists of clinical events (eg, medication list), abbreviations, and incomplete and short sentences, unduly lowering the readability score.

One measurement that tailors to the medical domain was proposed by Kim H et al [22]. This method compared surface text, syntactic, and semantic differences to predefined easy and difficult documents and reported normalized scores instead of grade levels. Another method for health text based on a naive Bayes classifier was developed [23]. The authors collected training documents from Web-based blogs, patient education documents, and medical journal articles. Vocabularies in these documents were used as features for the classifier. Both of the methods relied on manually curated documents. Therefore, different choices in constructing the sets might result in variation in the scores or classification results. Moreover, the classifier was limited, as it assigned only 3 categories—easy, intermediate, and difficult, and did not assign a grade-level scale. Furthermore, the reference document sets were not available.

Less research has been conducted on whether the readability grade levels predicted by these formulas or computational models agree with actual users’ perceptions of text difficulty. The objective characteristics are shown to not always align with user perceptions in other research fields. In one study, user perceptions of computer manufacturers’ websites were different from content analysis tools [41]. In this work, we explored the relationship between users’ perceptions of text difficulty and the readability formulas’ output.

We evaluated FKGL and other widely used traditional readability metrics. These metrics usually hinged on a few textual characteristics and did not take into account the domain of the text. We also explored the effectiveness of the existing readability formulas on predicting the users’ *perceptions* of difficulty. We hypothesized that the perceived readability of technical documents on complex topics was dependent on the domain of the text, not an absolute measure of the difficulty of a piece of text.

## Methods

### Overview

We evaluated existing metrics for assessing EHR readability and investigated their utility in EHR notes. We used the open-source Java library Flesh 2.0 [42] to calculate FKGL. In addition, we used the same program to calculate the number of sentences, words, and syllables, and then applied the other 2 formulas (SMOG and GFI). In the following sections, we first describe the data we used for evaluation, followed by an analysis of this corpus.

### Data

We collected documents about diabetes from 2 different resources: English Wikipedia (denoted as wiki) and deidentified EHR notes (denoted as med). In wiki documents, we traversed from the Diabetes category. The EHR notes were selected using the International Classification of Diseases, Ninth Revision, code range 250.00 to 250.93. The 2 sources provided a contrast between texts aimed at the general audience and those written with health care professionals in mind. The statistics of this collection is shown in Table 1 under the columns labeled “all.”

Diabetes is a common disease that we can expect a large body of readers to be aware of and can provide reasonable judgments on readability. This is especially important in the EHR collection because randomly selected EHR notes may contain information about rare conditions, which can confuse the readers. The common theme of the content in the 2 sources also helps address the problem of variations of a user’s knowledge in different areas. By constraining to a single condition, we can limit the confounding effect of a user’s different levels of familiarity in different areas.

**Table 1:** Document collection statistics.

Genre	Documents		Sentences		Tokens		FKGL^c^	
	All^a^	Paired^b^	All	Paired	All	Paired	All	Paired
Wiki	140	58	5703	1084	142, 106	23, 185	7.33–21.85	7.33–17.82
Med	242	133	8715	4232	120, 315	57, 655	6.48–15.76	6.99–15.76

^a^Columns labeled “all” include all documents.

^b^Columns labeled “paired” include only documents where another one with a similar length and FKGL score is also available.

^c^FKGL: Flesch-Kincaid Grade Level.

### Amazon Mechanical Turk Annotators

To validate one of the most frequently used readability formulas, FKGL, we paired analogous documents in our collection to ask Amazon Mechanical Turk (AMT) users to compare them. Specifically, documents were paired so that they had similar lengths (within 50-token difference) and comparable readability levels according to FKGL (within 0.5 grade level). The statistics on documents that were paired are shown in Table 1 under the columns labeled “paired.”

**Figure 1:**
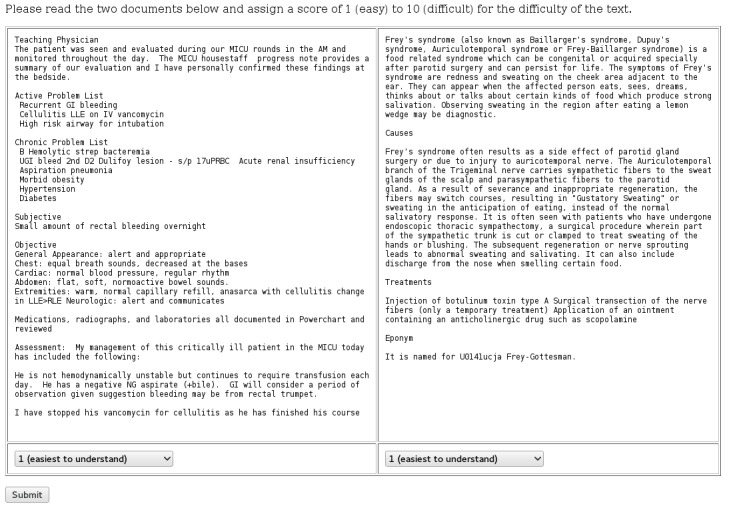
Screenshot of the interface for the Amazon Mechanical Turk (AMT) users.

We recruited 15 AMT subjects to read and rate pairs of documents. The readers were screened to have English as their native language and be AMT master workers. Three readers had a high school diploma, 7 had an associate degree, 4 had a Bachelor’s degree, and 1 did not report education level. Each reader was presented with 20 randomly selected pairs of documents side by side on the computer screen. The 20 document pairs consisted of 5 pairs of wiki documents, 5 pairs of med documents, and 10 pairs of mixed-genre documents. The readers were requested to rate the readability of the documents on a scale from 1 (easiest to understand) to 10 (most difficult to understand). Each reader was given 6 hours to complete the task, and was not explicitly prohibited from using external resources. On average, they finished the assignment in 1 hour. Figure 1 is a screenshot of the interface with a mixed-genre pair.

### Corpus Analysis

#### Readability and User Rating Distributions

We first analyzed the empirical distribution of AMT users’ ratings on the text difficulty and compared it with the empirical distribution of the readability formulas’ scores.

#### Correlation Between AMT Users

We next measured correlations between different AMT users. For each user, all the documents that he or she provided a rating were collected. Since the document pairs were randomly assigned, in general no 2 users worked on an identical set of documents. Only a subset of the documents were rated by any 2 users. On average, a document was rated by 2.3 users. Between 2 users, 8.6 documents were on average rated by both.

We calculated correlations for a user’s and any other user’s ratings on the documents that were rated by both. The average for each user was obtained by first transforming the correlations by Fisher z-transformation, and then back-transformed [43]. Document genres were not separated in the calculation; otherwise, it would result in too few instances.

#### Correlation Between AMT User and Readability Formulas

To evaluate traditional readability formulas’ applicability in technical documents, correlations between each AMT user’s ratings and the 3 readability formulas were measured separately for the wiki and med genres. The average over each user’s correlations were also obtained by Fisher z-transformation.

#### Differences in Users’ Perceived Difficulty

To validate the generalizability of FKGL to different genres of text, we tested whether users perceived a difference when the readability scores were similar. The AMT users in our experiments were presented with documents of comparable difficulty (within a difference of 0.5) according to FKGL and of similar length (within 50-token difference). We tested the statistical significance of the difference between the difficulty values assigned by the users to 2 similar documents, separately for wiki, med, and mixed pairs. Two statistical tests are employed—Wilcoxon signed-rank test and Kolmogorov-Smirnov test.

We also tested the generalizability of 2 other formulas using the same procedure. Among all of the document pairs, we selected the subset of document pairs in which the SMOG scores were within 0.5 between each pair. The same process was repeated using GFI scores.

Furthermore, we explored the disparity in users’ perceived difficulty when a readability formula reported a difference between 2 documents. For each user, we generated pairs of documents from all of the documents he or she rated and then removed the pairs that were presented during the AMT work session. These document pairs were separated into 3 types based on the genres of the documents, as in the previous experiments.

#### Correlation Between Readability Formulas

Since FKGL, SMOG, and GFI all involved similar variables (sentence length in words or polysyllabic words, word length), we examined the correlations between different readability formulas on the 2 genres of text in our dataset. Many studies adopted more than one of the traditional formulas to ascertain readability grade level on documents intended for patient consumption [44-49]. Analyzing the formulas’ correlations would inform us of this approach’s utility.

#### Word Usage

We compared the word usage patterns in the 2 genres of text by examining the common words. First, words in both med and wiki sources were ordered by the frequency in which they appeared in their respective genre. Then, the common words that were in both genres of text in the top frequently used words were counted. The shared vocabulary size might reveal a difference in word usage in different text genres.

#### Impact of Medical Concepts

Medical jargon is one of the barriers for the patient to understand health information. The eligibility criteria in clinical trials are found to be too difficult for the average American population, mainly due to the frequent use of technical jargon [50]. One study has shown that linking medical terms in EHR notes to Wikipedia pages can improve patient’s comprehension [51]. Moreover, many methods have been proposed to identify important or potentially unfamiliar medical terms [52,53].

We explored the effects of the medical concepts by measuring the correlation between users’ ratings and the number of concepts. Medical concepts were identified by running MetaMap [54] and excluding concepts from the following semantic groups and types: Activities & Behaviors, Concepts & Ideas, Geographic Areas, Objects, Occupations, Organizations, Age Group, Animal, Family Group, Group, Human, Patient or Disabled Group, Population Group, Professional or Occupational Group, Educational Activity, Health Care Activity, and Research Activity. These semantic groups and types usually do not contain technical medical jargon, and are uncommon in EHR notes. We also excluded Anatomical Structure because in our dataset almost all terms in this category were “body,” with the rest being such common body parts as “head” that would not pose difficulty for an average reader.

## Results

### Readability and User Rating Distributions

**Figure 2:**
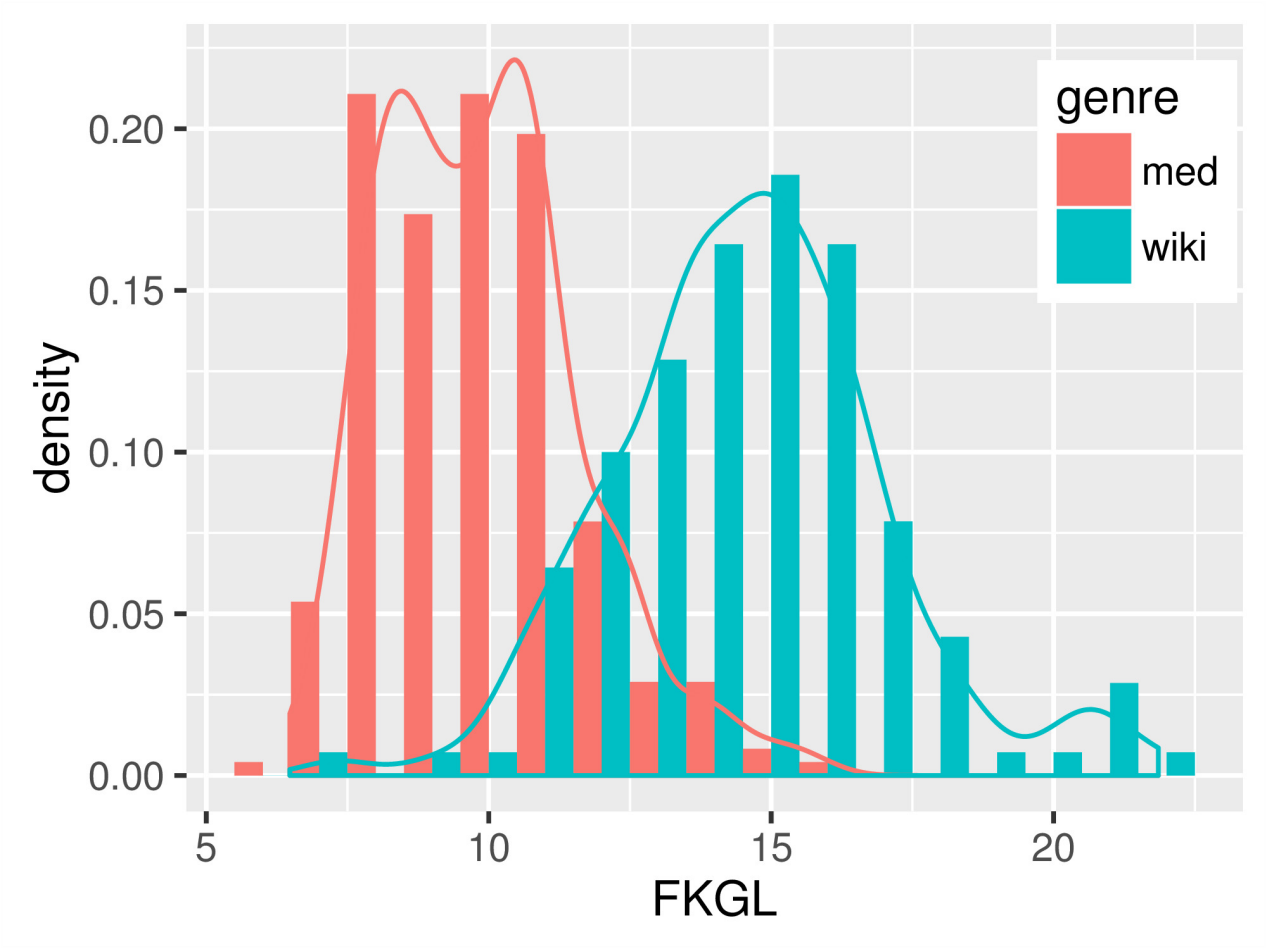
Histogram of Flesch-Kincaid Grade Level (FKGL).

**Figure 3:**
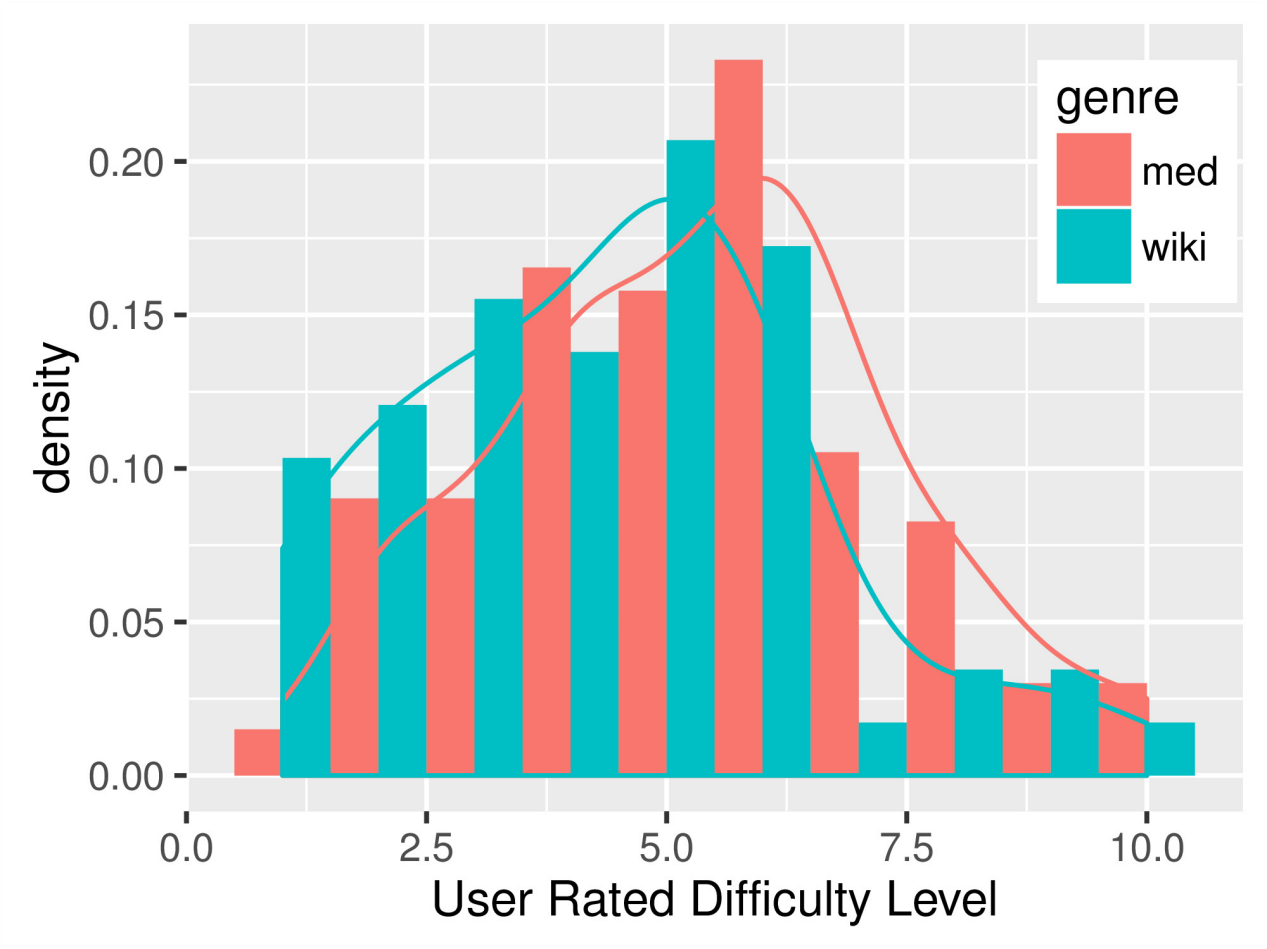
Histogram of Amazon Mechanical Turk (AMT) users’ ratings.

Empirical distributions of the FKGL readability scores and users’ ratings are shown in Figures 2 and 3. The FKGL histograms (Figure 2) on the 2 genres have clear distinctions. However, contrary to the general belief that EHR notes are more difficult to read, the histogram on the med data peaks to the left of the wiki data histogram. The users’ ratings (Figure 3), although to a smaller degree, show a higher difficulty level for the med than for the wiki data.

Table 2 shows the average score of each readability formula and the AMT users’ ratings. All of the 3 readability scores suggested that the technical EHR notes were significantly easier than lay language wiki articles, whereas the AMT users rated the opposite—wiki articles were 21.31% harder than EHR notes.

These results suggested that although FKGL might distinguish the readability of different genres, its counterintuitive predictions could lead to underestimation of difficulty levels on highly complex documents.

**Table 2:** Average readability score and users’ ratings.

Genre	Average score or rating			
	FKGL^a^	SMOG^b^	GFI^c^	AMT^d^ user rating
Wiki	14.75	11.07	12.33	4.41
Med	9.87	8.74	8.16	5.35
Difference^e^ (%)	−33.09	−21.03	−33.76	21.31
*P* value	<.001	<.001	<.001	.002

^a^FKGL: Flesch-Kincaid Grade Level.

^b^SMOG: Simple Measure of Gobbledygook.

^c^GFI: Gunning-Fog Index.

^d^AMT: Amazon Mechanical Turk.

^e^All differences in scores between the wiki and med genres were statistically significant at level *P*=.01 (Mann-Whitney *U* test). The second to last row shows that the percentage med score was higher than the percentage wiki score.

### Correlation Between AMT Users

Table 3 summarizes the correlations between 2 users’ ratings. Most users showed moderate or strong correlation with other users, suggesting that the AMT users’ perceptions of difficulty were congruous among themselves.

**Table 3:** Average correlations between a user and everyone else.

Average correlation	No. of users
<0.4	3
0.4–0.6	5
>0.6	7

### Correlation Between AMT User and Readability Formulas

Table 4 shows the average correlation coefficients between an AMT user’s ratings and the 3 readability formulas’ output. All the correlations were very low, especially in the med category. The SMOG and user rating correlation on wiki data, although slightly higher than that of FKGL and GFI, was barely moderate. The low correlations suggested that users’ perceived difficulty levels were inconsistent with the readability formulas’ predictions. For example, one user consistently assigned low difficulty levels to documents with FK scores 12–16. However, another user’s scores for documents with FK levels approximately 13.5 varied considerably. In contrast, the difficulty perceptions among different users were highly consistent (Table 3).

**Table 4:** Average correlation between users’ ratings and readability formulas.

Readability formula	Wiki	Med
FKGL^a^	0.1758	0.2999
SMOG^b^	0.4134	0.1024
GFI^c^	0.2695	0.1272

^a^FKGL: Flesch-Kincaid Grade Level.

^b^SMOG: Simple Measure of Gobbledygook.

^c^GFI: Gunning-Fog Index.

This pattern of inconsistency highlighted the inadequacy of these formulas’ utility in measuring EHR readability. It also highlighted their weakness in testing readability of documents of complex topics such as medicine, as they were developed to help users in the education community to gauge text difficulty below 12 grade. All 3 formulas relied on word counts and sentence counts to estimate text readability. The implicit assumption that longer words were more difficult, however, could often be violated. For instance, abbreviations that were not normally used outside the medical domain, such as “CHF” (Congestive Heart Failure) and “EKG” (electrocardiogram), were prevalent in EHR notes, without full definitions. Because these short abbreviations often comprised very few, if any, syllables, they would have exactly the same impact on the readability score as did the common stop words such as “the.” However, the abbreviations were obviously one of the barriers for a patient to understanding an EHR note. Furthermore, many abbreviations were ambiguous. For example, “MI” can be the shorthand for both “myocardial infarction” and “myocardial ischemia,” 2 different clinical conditions. In fact, disambiguating these abbreviations has been actively studied [55,56]. Finally, SMOG and GFI’s use of polysyllabic words could also exacerbate the problems with abbreviations. For example, “COPD” might be considered a 1-syllable word in calculating FKGL, but it would make no contribution to the calculation of SMOG or GFI.

### Differences in Users’ Perceived Difficulty

When 2 documents of similar length and FKGL score were shown together, the ratings assigned by the AMT users exhibited different patterns depending on the genres of the 2 documents. Using a Wilcoxon signed-rank test, the *P* values are displayed in Table 5 under “Wilcoxon signed-rank test.”

**Table 5:** Statistical significance of difference in AMT users’ perceived difficulty between documents of similar Flesch-Kincaid Grade Level.

Genre of pair	*P* value	
	Wilcoxon signed-rank test	Kolmogorov-Smirnov test
Wiki	.80	.95
Med	.25	.80
Mixed	<.001	<.001

The *P* values for a pair of same-genre documents showed that the users’ assignments were not significantly different, consistent with the traditional formula’s assessment. However, the *P* value for a pair of documents from different genres indicated that despite being assessed at similar difficulty, actual users perceived them as significantly different in terms of readability. Kolmogorov-Smirnov test (Table 5) also showed the same trend.

The same tests, when repeated on a subset of document pairs whose SMOG or GFI score difference was within 0.5, confirmed that they were not generalizable to different text domains. Detailed significance test results are displayed in Multimedia Appendix 2.

**Figure 4:**
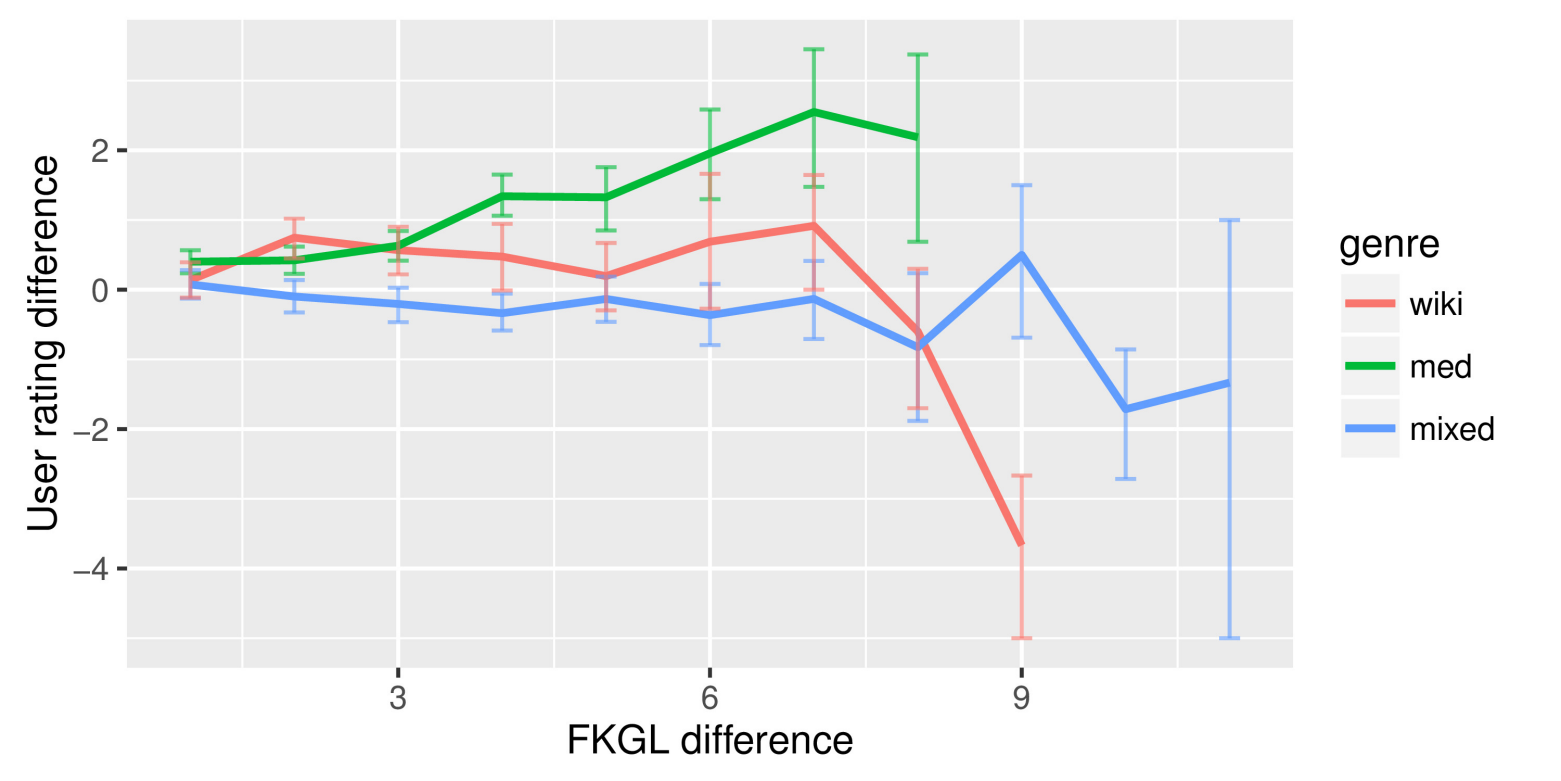
Average user’s rating difference on 2 documents of different Flesch-Kincaid Grade Level (FKGL) scores. Error bars are bootstrapped 95% CI.

AMT users’ perceptions of difficulty varied depending on the genre of text, even though a readability formula shows no difference. We then explored the disparity in users’ perceived difficulty when a readability formula reported a difference between 2 documents. Figure 4 shows the average difference in users’ ratings on a pair of documents with varying differences in FKGL scores.

For a pair of EHR notes, as the difference in FKGL scores widened, AMT users’ rating difference also gradually increased. However, the users’ ratings were limited to at most 2.5 levels difference even for large FKGL differences. For a pair of Wikipedia documents, AMT users’ rating difference varied slightly within 1 level. These patterns suggested that in a wide range of FKGL scores, users’ ratings did not agree with FKGL.

A similar trend was present in a mixed pair of documents: AMT users’ rating difference stayed close to 0. The limited difference in AMT users’ ratings indicated that FKGL scores did not align well with user perceptions across different genres.

### Correlation Between Readability Formulas

The correlation coefficients between different formulas confirmed that all 3 formulas were strongly correlated on our dataset regardless of text genre, consistent with the findings from previous studies [57,58]. Detailed plots and table showing the correlation are displayed in Multimedia Appendix 3. The substantial correlation implied that there was limited utility in employing multiple formulas, especially those relying on word and sentence lengths, to reduce potential bias of the individual ones when assessing text readability, as is often done in research studies [44-47,59].

### Word Usage

In 2 similar corpora, the N most frequent words from each corpus would be similar. Therefore, the number of common words would increase at approximately the same rate as more frequent words were examined from the 2 corpora. Significant deviations from this pattern were indications of different word usage patterns. As shown in Figure 4, in our set of diabetes documents, the rate of increase in common words between wiki and med documents was significantly smaller (at the level *P*<.001) than 1 (shown as the solid line in the figure). This suggested that the word usage patterns in the technical (med) and lay language (wiki) documents on the same topic were different.

**Figure 5:**
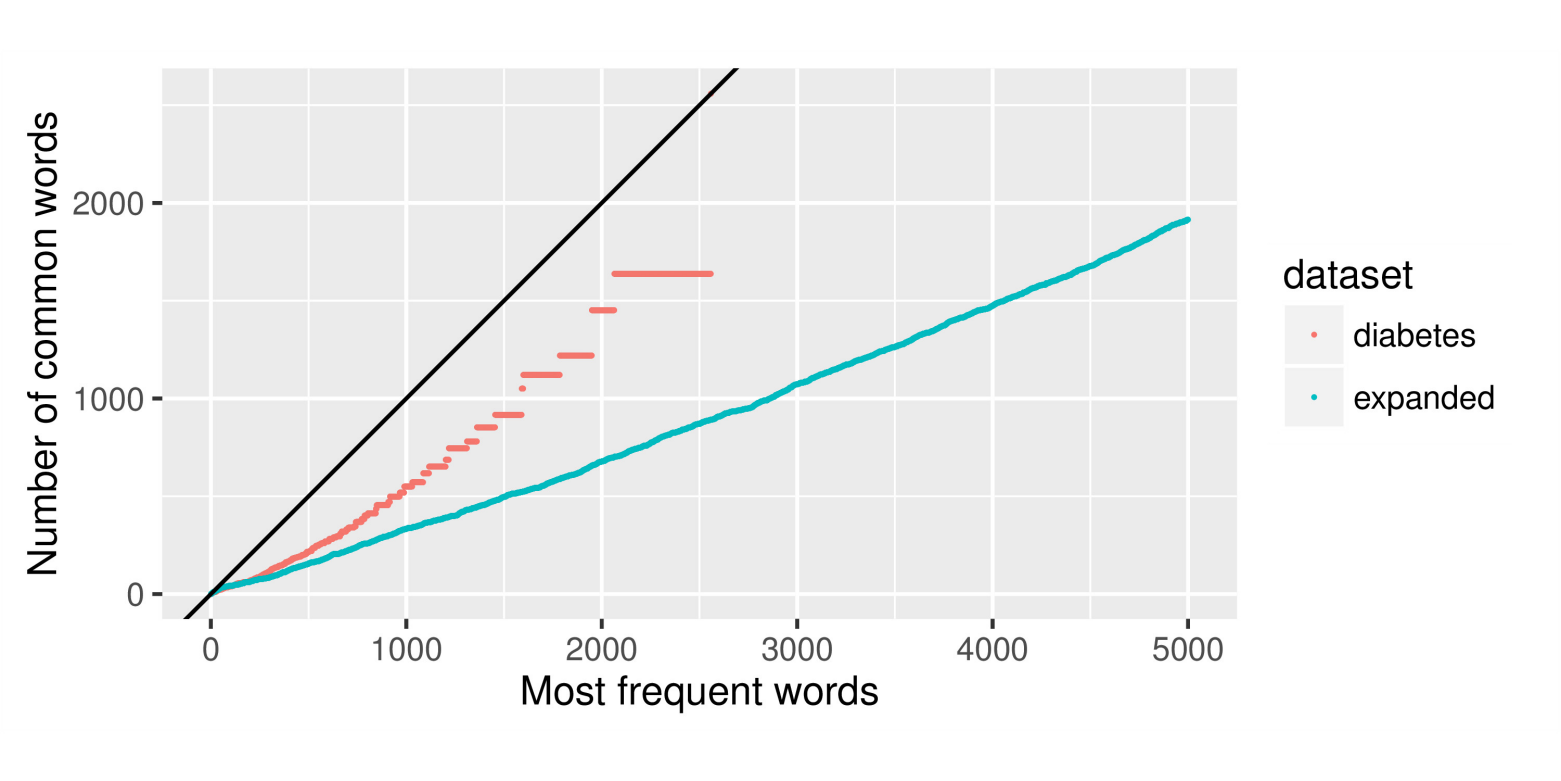
Common words in the med and wiki genre texts.

Expanding to more topics, we built the same word frequency statistic in all Wikipedia articles and about 100,000 EHR notes. Shown in Figure 5 as the “expanded” collection, the slope of common word count was also significantly smaller than 1 (at the level *P*<.001).

### Impact of Medical Concepts

The correlation coefficients between the number of medical concepts and user difficulty ratings, shown in Table 6, were measured for each user and averaged. The average correlation was again obtained by Fisher *z*-transformation. Correlations with unique concepts were slightly higher than correlations with all concepts in both med and wiki texts. More unique medical concepts were likely to result in more cognitive load for a user to comprehend. It was also worth noting that EHR notes showed a lower level of correlation than the Wikipedia documents. This could be in part attributed to the multitude of complexities of EHR notes not limited to the abundance of technical jargon. Writing style such as choice of words and textual cohesion might also account for some of the variances in the difficulty in perceptions of EHR notes. In spite of the differences, these correlations suggested that medical jargon was a substantial contributor to readers’ perceived difficulty of both genres of text.

**Table 6:** Average correlations between users’ ratings and number of medical concepts.

Number of medical concepts	Wiki	Med
Number of all concepts	0.4434	0.3987
Number of unique concepts	0.5041	0.4329

## Discussion

### Principal Findings

We evaluated 3 widely used readability formulas’ predictions of text difficulty and their correlation with users’ perceived difficulty. Our results showed that although the formulas’ predictions were highly correlated, they did not align well with user perceptions. Furthermore, despite being graded at similar levels by a readability formula, documents of different genres were still perceived with different difficulty.

Numerous studies have used the traditional readability formulas to evaluate a variety of information sources for patients. Many conclude that the materials intended for patients to improve understanding of their health conditions are too complex, exceeding the recommended grade levels for this purpose. Some also suggest areas of improvements to help align the documents to recommendation levels.

Unlike these studies that focus on documents mainly for patient consumption (patient handouts, education materials, Web-based information sources), we aimed to provide insight into users’ *perceptions* of EHR notes readability. EHR notes are foremost a tool for physician communication, and a large portion of them are not originally written with easy patient comprehension in mind. However, they are shown to be beneficial to the patients. As more institutions allow patients access to their own EHR records, patients are also interested in reading them. Research has shown that patients may need help in understanding them. An accurate readability metric for the EHR notes can encourage physicians to write notes in a simpler language. It may make patient portals more useful. For example, it can be helpful in EHR record presentation by highlighting the easier ones that are within a patient’s reading capabilities and providing comprehension assistance on the difficult ones. Such a metric may also enable the assistance tools to locate education materials that are easier to read than the EHR notes.

We noted that readability was not the only factor affecting patients’ comprehension of EHR notes. For example, reader interest and motivation have been pointed out in the literature to be a factor contributing to comprehension [60]. In a more realistic scenario where patients read their own EHR notes, they are likely to be motivated and show interest in knowing their own health conditions. Comprehension may also depend on a user’s existing knowledge of the subject matter. Since we evaluated on many anonymous AMT users, the bias in individual prior knowledge was reduced due to such a random selection of participants. Nonetheless, higher readability could facilitate patient comprehension. Existing formulas are widely used in the health domain, but our analysis suggested that they were not accurate when applied on complex documents. A better metric should incorporate features beyond simple word and sentence length, such as the complexity of the concepts involved in the document.

Developments in computer science have led to new methods that utilize statistical language modeling and machine learning to predict text readability. For example, readability levels are modeled as a linear combination of a unigram language model and a normal distribution-based sentence length model [61]. This approach is expanded by introducing smoothing into the language models [62]. A Support Vector Machine model to assess text readability is proposed in [63] to learn from features including syntactic information such as noun phrases, traditional readability formulas, and language models.

However, language model-based approaches do not receive so widely an adoption in the medical domain as the traditional formulas. This can be in part due to the need to learn a language model, a much more involved task than using a formula. FKGL and other formulas, on the contrary, are readily available, free of charge, and easy to use [64]. We will explore these new models’ performance in differentiating user perceptions of difficulty in the future.

### Limitations

Our analysis was based on recruiting volunteers from AMT to evaluate readability of EHR records. Having patients directly perform this evaluation might represent a more realistic user experience. The patient, through his or her interactions with a health care professional, might have a better context than an AMT user to rate his or her own record’s readability. In our study, to mitigate the problem, we selected documents based on a common condition.

Our sample documents were from one condition, selected to provide some context so that users would not be surprised by an unfamiliar topic. Thus, the user’s prior knowledge might affect their ratings of text difficulty.

There are several other avenues we plan to pursue in future work. Clustering users based on their pre-existing knowledge may reveal readability formulas’ differing capabilities in predicting users’ perceptions of difficulty for different populations. We also plan to develop new methods that can better capture the readability of complex technical documents so that both health care providers and patients can benefit from focusing first on EHR notes that are at an appropriate difficulty level.

### Conclusions

Studies have shown that providing patients with access to their own EHR notes may lead to improved health care outcomes. Measuring the readability of the EHR notes is an important step toward making the highly complex and technical narratives accessible to the patients. Despite being widely used in the health care domain, existing readability formulas are not thoroughly validated for their appositeness in this domain. In this study, we evaluated several such formulas’ abilities in predicting *perceptions* of difficulty in health-related text from Wikipedia and EHR notes. We collected AMT users’ ratings on text difficulty from these 2 different genres. Word usage in the 2 genres differed significantly despite their sharing a common topic. We found that the readability formulas’ predictions did not align with perceived difficulty in either text genre. Furthermore, there was significant difference in the user’s perceived difficulty in the general English and medical language when similar scores were predicted by readability formulas. Therefore, the widely used and highly correlated FKGL, SMOG, and GFI readability scales did not show adequate agreement with human ratings, and thus were not appropriate to assess the readability of EHR notes.

## Appendix

Multimedia Appendix 1 Details of the readability formulas evaluated.

Multimedia Appendix 2 Statistical significance of the difference in AMT users’ perceived difficulty between documents of similar SMOG or GFI levels.

Multimedia Appendix 3 Correlations of grade levels from different readability formulas.

## Abbreviations

AMT: Amazon Mechanical Turk
EHR: electronic health records
FKGL: Flesch-Kincaid Grade Level
GFI: Gunning-Fog Index
SMOG: Simple Measure of Gobbledygook

## References

[1] Institute of Medicine, Committee on Quality of Health Care in America (2001). Crossing the quality chasm: a new health system for the 21st century.

[2] Greene J, Hibbard JH (2012). Why does patient activation matter? An examination of the relationships between patient activation and health-related outcomes. J Gen Intern Med.

[3] White A, Danis M (2013). Enhancing patient-centered communication and collaboration by using the electronic health record in the examination room. J Am Med Assoc.

[4] Delbanco T, Walker J, Bell SK, Darer JD, Elmore JG, Farag N, Feldman HJ, Mejilla R, Ngo L, Ralston JD, Ross SE, Trivedi N, Vodicka E, Leveille SG (2012). Inviting patients to read their doctors' notes: a quasi-experimental study and a look ahead. Ann Intern Med.

[5] Wiljer D, Bogomilsky S, Catton P, Murray C, Stewart J, Minden M (2006). Getting results for hematology patients through access to the electronic health record. Can Oncol Nurs J.

[6] Tang PC, Lansky D (2005). The missing link: bridging the patient-provider health information gap. Health Aff (Millwood).

[7] Mossanen M, True LD, Wright JL, Vakar-Lopez F, Lavallee D, Gore JL (2014). Surgical pathology and the patient: a systematic review evaluating the primary audience of pathology reports. Hum Pathol.

[8] Keselman A, Slaughter L, Smith CA, Kim H, Divita G, Browne A, Tsai C, Zeng-Treitler Q (2007). Towards consumer-friendly PHRs: patients' experience with reviewing their health records. AMIA Annu Symp Proc.

[9] Keselman A, Smith CA (2012). A classification of errors in lay comprehension of medical documents. J Biomed Inform.

[10] Pyper C, Amery J, Watson M, Crook C (2004). Patients' experiences when accessing their on-line electronic patient records in primary care. Br J Gen Pract.

[11] Mák G, Smith FH, Leaver C, Hagens S, Zelmer J (2015). The effects of web-based patient access to laboratory results in british columbia: a patient survey on comprehension and anxiety. J Med Internet Res.

[12] Chapman K, Abraham C, Jenkins V, Fallowfield L (2003). Lay understanding of terms used in cancer consultations. Psychooncology.

[13] Lerner EB, Jehle DV, Janicke DM, Moscati RM (2000). Medical communication: do our patients understand?. Am J Emerg Med.

[14] Zeng QT, Tse T, Divita G, Keselman A, Crowell J, Browne AC, Goryachev S, Ngo L (2007). Term identification methods for consumer health vocabulary development. J Med Internet Res.

[15] Zielstorff RD (2003). Controlled vocabularies for consumer health. J Biomed Inform.

[16] Patrick TB, Monga HK, Sievert ME, Houston HJ, Longo DR (2001). Evaluation of controlled vocabulary resources for development of a consumer entry vocabulary for diabetes. J Med Internet Res.

[17] Kutner M, Greenburg E, Jin Y, Paulsen C NCES.

[18] Boles CD, Liu Y, November-Rider D (2016). Readability levels of dental patient education brochures. J Dent Hyg.

[19] Huang G, Fang CH, Agarwal N, Bhagat N, Eloy JA, Langer PD (2015). Assessment of online patient education materials from major ophthalmologic associations. JAMA Ophthalmol.

[20] Grossman SA, Piantadosi S, Covahey C (1994). Are informed consent forms that describe clinical oncology research protocols readable by most patients and their families?. J Clin Oncol.

[21] Redish J (2000). Readability formulas have even more limitations than Klare discusses. ACM J Comput Doc.

[22] Kim H, Goryachev S, Rosemblat G, Browne A, Keselman A, Zeng-Treitler Q (2007). Beyond surface characteristics: a new health text-specific readability measurement. AMIA Annu Symp Proc.

[23] Leroy G, Miller T, Rosemblat G, Browne A (2008). A balanced approach to health information evaluation: a vocabulary-based naïve Bayes classifier and readability formulas. J Am Soc Inf Sci.

[24] Klare GR (1974). Assessing readability. Read Res Q.

[25] Flesch R (1948). A new readability yardstick. J Appl Psychol.

[26] McLaughlin GH (1969). SMOG grading-a new readability formula. Journal of reading.

[27] Gunning R (1968). The technique of clear writing.

[28] Williamson JML, Martin AG (2010). Analysis of patient information leaflets provided by a district general hospital by the Flesch and Flesch-Kincaid method. Int J Clin Pract.

[29] Wilson M (2009). Readability and patient education materials used for low-income populations. Clin Nurse Spec.

[30] Woodmansey K (2010). Readability of educational materials for endodontic patients. J Endod.

[31] Cheng C, Dunn M (2015). Health literacy and the Internet: a study on the readability of Australian online health information. Aust N Z J Public Health.

[32] Eltorai AE, Ghanian S, Adams Jr CA, Born CT, Daniels AH (2014). Readability of patient education materials on the American association for surgery of trauma website. Arch Trauma Res.

[33] Khurana RN, Lee PP, Challa P (2003). Readability of ocular medication inserts. J Glaucoma.

[34] Wallace LS, Keenum AJ, Roskos SE, Blake GH, Colwell ST, Weiss BD (2008). Suitability and readability of consumer medical information accompanying prescription medication samples. Patient Educ Couns.

[35] Tarnowski KJ, Allen DM, Mayhall C, Kelly PA (1990). Readability of pediatric biomedical research informed consent forms. Pediatrics.

[36] Paasche-Orlow MK, Taylor HA, Brancati FL (2003). Readability standards for informed-consent forms as compared with actual readability. N Engl J Med.

[37] Wu DT, Hanauer DA, Mei Q, Clark PM, An LC, Proulx J, Zeng QT, Vydiswaran VG, Collins-Thompson K, Zheng K (2016). Assessing the readability of ClinicalTrials.gov. J Am Med Inform Assoc.

[38] Thomas GR, Eng L, de Wolff JF, Grover SC (2013). An evaluation of Wikipedia as a resource for patient education in nephrology. Semin Dial.

[39] Azer SA, AlSwaidan NM, Alshwairikh LA, AlShammari JM (2015). Accuracy and readability of cardiovascular entries on Wikipedia: are they reliable learning resources for medical students?. BMJ Open.

[40] Wang L, Miller MJ, Schmitt MR, Wen FK (2013). Assessing readability formula differences with written health information materials: application, results, and recommendations. Res Social Adm Pharm.

[41] Lee S, Lee W, Kim H, Stout PA (2004). A comparison of objective characteristics and user perception of web sites. J Interact Advert.

[42] Flesh.sourceforge.

[43] Silver NC, Dunlap WP (1987). Averaging correlation coefficients: Should Fisher's z transformation be used?. J Appl Psychol.

[44] Hansberry DR, Agarwal N, Gonzales SF, Baker SR (2014). Are we effectively informing patients? A quantitative analysis of on-line patient education resources from the American Society of Neuroradiology. AJNR Am J Neuroradiol.

[45] Hansberry DR, Agarwal N, Baker SR (2015). Health literacy and online educational resources: an opportunity to educate patients. AJR Am J Roentgenol.

[46] Vargas CR, Koolen PG, Chuang DJ, Ganor O, Lee BT (2014). Online patient resources for breast reconstruction: an analysis of readability. Plast Reconstr Surg.

[47] Vargas CR, Chuang DJ, Ganor O, Lee BT (2014). Readability of online patient resources for the operative treatment of breast cancer. Surgery.

[48] Taki S, Campbell KJ, Russell CG, Elliott R, Laws R, Denney-Wilson E (2015). Infant feeding websites and apps: a systematic assessment of quality and content. Interact J Med Res.

[49] Piñero-López MÁ, Modamio P, Lastra CF, Mariño EL (2016). Readability analysis of the package leaflets for biological medicines available on the internet between 2007 and 2013: an analytical longitudinal study. J Med Internet Res.

[50] Kang T, Elhadad N, Weng C (2015). Initial readability assessment of clinical trial eligibility criteria. AMIA Annu Symp Proc.

[51] Polepalli RB, Houston T, Brandt C, Fang H, Yu H (2013). Improving patients' electronic health record comprehension with NoteAid. Stud Health Technol Inform.

[52] Elhadad N (2006). Comprehending technical texts: predicting and defining unfamiliar terms. AMIA Annu Symp Proc.

[53] Zheng J, Yu H (2015). Key concept identification for medical information retrieval.

[54] Aronson AR, Lang F (2010). An overview of MetaMap: historical perspective and recent advances. J Am Med Inform Assoc.

[55] Xu H, Stetson PD, Friedman C (2007). A study of abbreviations in clinical notes. AMIA Annu Symp Proc.

[56] Kim Y, Hurdle J, Meystre SM (2011). Using UMLS lexical resources to disambiguate abbreviations in clinical text. AMIA Annu Symp Proc.

[57] Štajner S, Evans R, Orasan C, Mitkov R (2012). What can readability measures really tell us about text complexity. Proceedings of the 8th International Conference on Language Resources and Evaluation.

[58] Van Oosten OP, Tanghe D, Hoste V (2010). Towards an improved methodology for automated readability prediction.

[59] Badarudeen S, Sabharwal S (2010). Assessing readability of patient education materials: current role in orthopaedics. Clin Orthop Relat Res.

[60] Baldwin RS, Peleg-Bruckner Z, McClintock AH (1985). Effects of topic interest and prior knowledge on reading comprehension. Read Res Q.

[61] Si L, Callan J (2001). A statistical model for scientific readability.

[62] Collins-Thompson K, Callan J (2004). A language modeling approach to predicting reading difficulty.

[63] Schwarm SE, Ostendorf M (2005). Reading level assessment using support vector machines and statistical language models.

[64] De Felippe N, Kar F (2015). Readability of information related to the parenting of a child with a cleft. Interact J Med Res.

