# Supplementary material for: Readability Formulas and User Perceptions of Electronic Health Records Difficulty: A Corpus Study
Source: J Med Internet Res. 2017 Mar 2;19(3):e59. doi: 10.2196/jmir.6962 (PMC5355629; doi:10.2196/jmir.6962)
Supplement: Multimedia Appendix 1 [file jmir_v19i3e59_app1.pdf]

Multimedia Appendix 1

Readability formulas evaluated in this study.

Flesch-Kincaid Grade Level (FKGL)

$$0.39 \times \frac{total\ words}{total\ sentences} + 11.8 \times \frac{total\ syllables}{total\ words} - 15.59$$

Simple Measure of Gobbledygook (SMOG)

$$1.0430 \times \sqrt{\frac{polysyllabic\ words}{sentences}} + 3.1291$$

Gunning-Fog Index (GFI)

$$0.4 \times \left(\frac{words}{sentences} + 100 \times \frac{polysyllabic\ words}{words} \right)$$
