# Supplementary material for: Readability Formulas and User Perceptions of Electronic Health Records Difficulty: A Corpus Study
Source: J Med Internet Res. 2017 Mar 2;19(3):e59. doi: 10.2196/jmir.6962 (PMC5355629; doi:10.2196/jmir.6962)
Supplement: Multimedia Appendix 2 [file jmir_v19i3e59_app2.pdf]

Multimedia Appendix 2

When readability is measured by FKGL, the AMT users show significant perceived difference in a document pair of mixed genres, whereas documents in the same genre do not exhibit significant difference (Table 5). This result is consistent with those presented in Table A2-1, where readability is measured by SMOG and GFI.

Table A2-1: Statistical significance of difference in AMT users' perceived difficulty between documents of similar SMOG or GFI levels.

Genre of Pair	SMOG		GFI	
	Sgn-Rank test	K-S test	Sgn-Rank test	K-S test
wiki	1	1	1	1
med	1	.999	.821	1
mixed	<.001	<.001	<.001	.003
