# Supplementary material for: Readability Formulas and User Perceptions of Electronic Health Records Difficulty: A Corpus Study
Source: J Med Internet Res. 2017 Mar 2;19(3):e59. doi: 10.2196/jmir.6962 (PMC5355629; doi:10.2196/jmir.6962)
Supplement: Multimedia Appendix 3 [file jmir_v19i3e59_app3.pdf]

Multimedia Appendix 3

Figures A3-1 and A3-2 show the correlations of SMOG and GFI measured separately against FKGL. Both plots show a positive linear trend between FKGL and the other formulas. Table A3-1 shows the correlation coefficients between two formulas.

Figure A3-1: Scatter plot of SMOG and GFI scores against FKGL on wiki genre text.

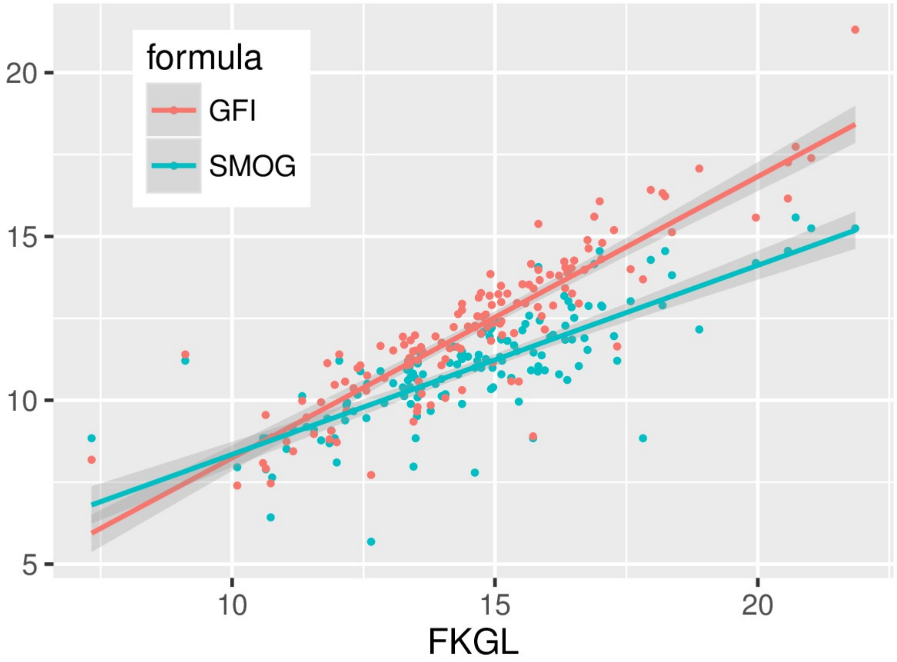

Figure A3-2: Scatter plot of SMOG and GFI scores against FKGL on med genre text.

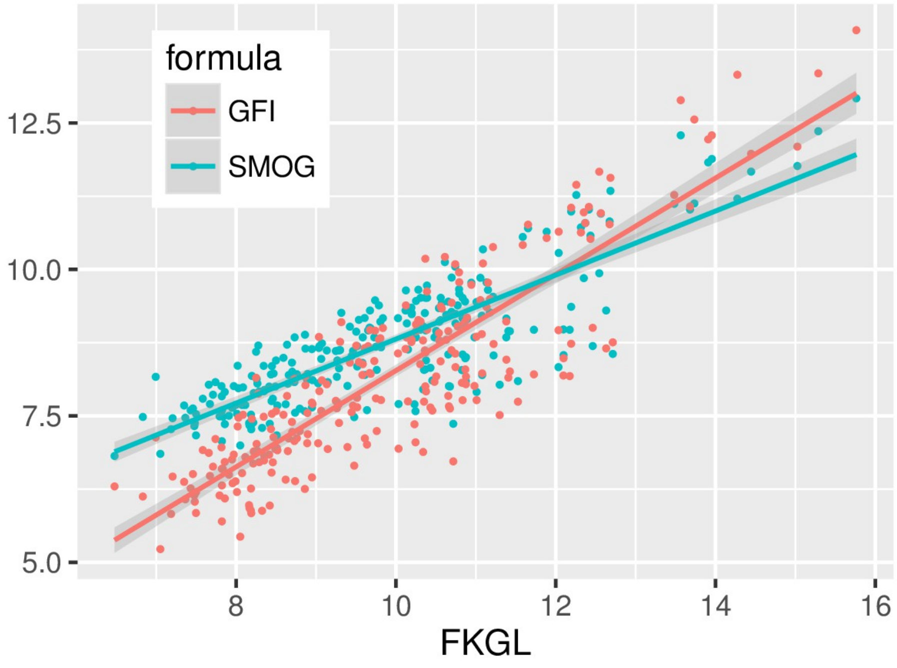

Table A3-1: Correlation coefficients between readability formulas. All correlations are significant (*P*<.001).

	wiki	med
FKGL-SMOG	0.8124	0.8428
FKGL-GFI	0.9191	0.8784
SMOG-GFI	0.8952	0.9696
